# Antidepressant use in bipolar disorder, a case report

**DOI:** 10.1192/j.eurpsy.2025.1048

**Published:** 2025-08-26

**Authors:** R. M. Brito Rey, R. Diler

**Affiliations:** 1 Psychiatry, Complejo Asistencial Universitario de Salamanca, Salamanca, Spain; 2 Psychiatry, UPMC, CABS, Pittsburgh, United States

## Abstract

**Introduction:**

Pediatric bipolar disorder is a severe and disabling condition that affects around 1–3% of youth worldwide. The typical clinical course consists of alternating episodes of depressed or elevated mood, with intervals of well-being. Studies have shown that the onset of bipolar disorder is frequently of depressive polarity, and the long-term affective morbidity is primarily depressive, even in patients who have received treatment. This indicates that achieving an adequate response to these mood states remains a challenge.

**Objectives:**

In relation to a case of a patient with pediatric bipolar disorder who received treatment with SSRIs in the CABS program, we reviewed the existing literature regarding the efficacy, safety, and tolerability of antidepressant medications in the pediatric population with bipolar disorder.

**Methods:**

We conducted a literature review of studies published in the past five years. We searched PubMed using the keywords “antidepressant,” “pediatric bipolar depression,” and “bipolar disorder.” We discuss the case of a 17-year-old patient undergoing treatment in the CABS program with a diagnosis of bipolar I disorder.

**Results:**

Studies have found that antidepressants are generally safe and well-tolerated. However, in bipolar disorders, antidepressants should be used in combination with mood stabilizers to reduce the risk of manic or hypomanic switching, especially in bipolar I disorder. The combination of olanzapine and fluoxetine has been found to be effective and has been approved by the FDA for both adults and adolescents with bipolar depression. SSRIs have shown effectiveness for pediatric bipolar depression in some studies, although the level of evidence is low. They are mainly used in cases of bipolar depression with comorbid anxiety. Bupropion is used off-label for ADHD in both pediatric and adult populations and is often prescribed for depression associated with bipolar disorder in adults. It is considered to have a lower risk of manic or hypomanic switch. Evidence for the use of ketamine in bipolar depression in adults remains unclear, particularly regarding long-term effects.

We present the case of a 17-year-old male with a diagnosis of bipolar I disorder who is undergoing treatment in the CABS program. He has a history of two hospitalizations and is currently being treated with aripiprazole. Escitalopram was added after he reported anxiety symptoms that aripiprazole was not addressing, resulting in a favorable response with no reported side effects.

**Conclusions:**

Antidepressants may play a role in some cases of pediatric bipolar depression, especially when anxiety symptoms are also present and do not respond to psychotherapeutic approaches. It is important to balance the risks and benefits of using antidepressants in this population. Close monitoring is recommended to assess response and possible side effects. Further research regarding the treatment of pediatric bipolar depression is needed.

**Disclosure of Interest:**

None Declared

